# Comparison of Metabolic Response to Colonic Fermentation in Lean Youth vs Youth With Obesity

**DOI:** 10.1001/jamanetworkopen.2023.12530

**Published:** 2023-05-09

**Authors:** Brittany Galuppo, Giuseppina Rosaria Umano, Zhongyao Li, Michelle Van Name, Stephanie L. Samuels, C. Lawrence Kien, Gary W. Cline, David A. Wagner, Emiliano Barbieri, Domenico Tricò, Nicola Santoro

**Affiliations:** 1 Touro College of Osteopathic Medicine, Middletown, New York, New York; 2 Department of Pediatrics, Yale School of Medicine, New Haven, Connecticut; 3 Department of the Woman, the Child, and General and Specialized Surgery, University of Campania, Luigi Vanvitelli, Naples, Italy; 4 Larner College of Medicine, University of Vermont, Burlington; 5 Department of Internal Medicine, Yale School of Medicine, New Haven, Connecticut; 6 Metabolic Solutions, Nashua, New Hampshire; 7 Scuola di Specializzazione in Pediatria Universita’ Federico II, Napoli, Italy; 8 Department of Clinical and Experimental Medicine, University of Pisa, Pisa, Italy; 9 Kansas University Medical Center, Kansas City; 10 Department of Medicine and Health Sciences, “V. Tiberio” University of Molise, Campobasso, Italy

## Abstract

**Importance:**

Pediatric obesity is a growing health care burden. Understanding how the metabolic phenotype of youth with obesity may modify the effect of intestinal fermentation on human metabolism is key to designing early intervention.

**Objective:**

To assess whether adiposity and insulin resistance in youth may be associated with colonic fermentation of dietary fibers and its production of acetate, gut-derived hormone secretion, and adipose tissue lipolysis.

**Design, Setting, and Participants:**

Cross-sectional study of youths aged 15 to 22 years with body mass index in the 25th to 75th percentile or higher than the 85th percentile for age and sex throughout the New Haven County community in Connecticut. Recruitment, studies, and data collection occurred from June 2018 to September 2021. Youths were assigned to a lean, obese insulin sensitive (OIS), or obese insulin resistant (OIR) group. Data were analyzed from April 2022 to September 2022.

**Exposure:**

Participants consumed 20 g of lactulose during a continuous 10-hour sodium d3-acetate intravenous infusion to measure the rate of appearance of acetate in plasma.

**Main Outcomes and Measures:**

Plasma was obtained hourly to measure acetate turnover, peptide tyrosine tyrosine (PYY), ghrelin, active glucagon-like peptide 1 (GLP-1), and free fatty acids (FFA).

**Results:**

A total of 44 youths participated in the study (median [IQR] age, 17.5 [16.0-19.3] years; 25 [56.8%] were female; 23 [52.3%] were White). Consequent to lactulose ingestion, there was a reduction of plasma FFA, an improvement of adipose tissue insulin sensitivity index, an increase in colonic acetate synthesis, and an anorexigenic response characterized by an increased plasma concentration of PYY and active GLP-1 and a reduction of ghrelin in the subgroups. Compared with the lean and OIS groups, the OIR group showed a less marked median (IQR) rate of acetate appearance (OIR: 2.00 [−0.86 to 2.69] μmol × kg^−1^ × min^−1^; lean: 5.69 [3.04 to 9.77] μmol × kg^−1^ × min^−1^; lean vs OIR *P* = .004; OIS: 2.63 [1.22 to 4.52] μmol × kg^−1^ × min^−1^; OIS vs OIR *P* = .09), a blunted median (IQR) improvement of adipose insulin sensitivity index (OIR: 0.043 [ 0.006 to 0.155]; lean: 0.277 [0.220 to 0.446]; lean vs OIR *P* = .002; OIS: 0.340 [0.048 to 0.491]; OIS vs OIR *P* = .08), and a reduced median (IQR) PYY response (OIR: 25.4 [14.8 to 36.4] pg/mL; lean: 51.3 [31.6 to 83.3] pg/mL; lean vs OIR *P* = .002; OIS: 54.3 [39.3 to 77.2] pg/mL; OIS vs OIR *P* = .011).

**Conclusions and Relevance:**

In this cross-sectional study, lean, OIS, and OIR youth demonstrated different associations between colonic fermentation of indigestible dietary carbohydrates and the metabolic response, with OIR youth showing minimal metabolic modifications as compared with the other 2 groups.

**Trial Registration:**

ClinicalTrials.gov Identifier: NCT03454828

## Key Points

**Question:**

What is the association of obesity and insulin resistance in youth with colonic fermentation of indigestible carbohydrates and its production of colon-derived acetate, gut-derived hormone secretion, and adipose tissue lipolysis?

**Findings:**

In this cross-sectional study of 44 participants, as compared with lean and obese insulin sensitive youth, obese insulin resistant youth showed a less marked rate of acetate appearance in plasma, blunted improvement of adipose tissue insulin sensitivity, greater insulin secretion, lower insulin clearance, and reduced anorexigenic hormone response after the ingestion of 20 g of lactulose, an indigestible dietary fiber.

**Meaning:**

These findings suggest that once insulin resistance occurs in the setting of obesity, the metabolic benefits that are usually seen with the consumption of dietary undigestible carbohydrates, such as fibers, may be lost, even as early as in adolescence.

## Teaser

This cross-sectional study compares metabolic response to colonic fermentation in lean youth and youth with obesity.

## Introduction

Pediatric obesity is a growing concern, affecting about 20% of children and adolescents in the US today.^1,2^ Obesity is a complex disease that results from the interaction between genetic background, behavior, and environment. Once individuals are affected, metabolic changes, such as insulin resistance (IR), worsen the condition and trigger a vicious cycle that leads to cardiometabolic complications.^3,4,5,6^

Indigestible carbohydrates such as fibers are often included in the dietary approach to combat obesity, as they improve metabolic outcomes and reduce adiposity.^7,8^ Although the mechanisms by which these nutrients act remain unknown, some studies suggest that they may exert their beneficial effects through the process of colonic fermentation.^9^ Colonic fermentation occurs when undigested carbohydrates reach the intestine and are metabolized by bacteria in the gut. This leads to the production of short chain fatty acids (SCFAs), the most abundant of which is acetate.^10^ A greater amount of complex carbohydrates in the diet ultimately leads to higher rates of colonic fermentation.^11^

We have recently completed a study to assess rates of colonic fermentation in youth with and without obesity.^12^ The plasma rate of appearance of acetate derived from colonic fermentation (Ra_acetate_) was measured before and after the ingestion of 20 g of lactulose for a total of 10 hours.^12^ We have shown that the rate at which acetate is produced after colonic fermentation is higher in lean youth than in youth with obesity and that it is associated with the rate of hepatic de novo lipogenesis.^12^ In this study, we further expand on those findings by describing the association of adipose tissue, insulin clearance, as well as peptide tyrosine tyrosine (PYY), ghrelin, and active glucagon-like peptide 1 (GLP-1) to colonic fermentation in youth. We also assess how adiposity and insulin resistance may be associated with the response of colonic fermentation on adipose tissue lipolysis, insulin clearance, and enteroendocrine secretion.

## Methods

### Study Cohort and Screening

Participants were recruited via study flyers that were posted publicly throughout the New Haven County community in Connecticut for this cross-sectional study. Recruitment, studies, and data collection occurred from June 2018 to September 2021. Eligibility inclusion criteria included: being aged 15 to 22 years and body mass index (BMI) 25th to 75th percentile or higher than the 85th percentile for age and sex. Exclusion criteria included: use of medication on a chronic basis, antibiotic use in the previous 3 months, pregnancy in girls, alcohol use, and dietary restrictions. Given the different metabolic features among different ethnicities and races, participants were asked for self-reported race and ethnicity. Of those enrolled in the study, 38 participants were part of previous studies to assess differences in acetate turnover after the ingestion of lactulose between lean youth and youth with obesity.^12^ The study was approved by the Yale University Human Investigations Committee in accordance with the Helsinki Declaration of 1975 as revised in 1983.^13^ Written consent from adults and parents and written assent from minors were obtained from all participants after full explanation of the study. This report follows the Strengthening the Reporting of Observational Studies in Epidemiology (STROBE) reporting guideline. Data were analyzed from April 2022 to September 2022.

The cohort was divided into 3 phenotypes, lean, obese insulin sensitive (OIS), and obese insulin resistant (OIR), according to BMI percentile and insulin sensitivity. Participants with BMI between the 25th and 85th percentile were categorized as a lean phenotype, and participants with a BMI higher than the 85th percentile were categorized as an obesity phenotype. Whole body insulin sensitivity index (WBISI) was used as a measure of insulin sensitivity and was calculated from the oral glucose tolerance test as reported by Matsuda.^14^ Youth with obesity were assigned to an OIS or OIR group according to the median WBISI of all participants with obesity (1.91), with the OIS and OIR classification falling above or below the median, respectively. Because of the statistically significant association between WBISI and homeostatic model assessment for insulin resistance (HOMA-IR) in this group, for 3 participants with obesity that did not complete an oral glucose tolerance test, HOMA-IR was used instead to determine insulin sensitivity for group assignment; if the HOMA-IR was greater than the median (calculated within the group with obesity), then they were considered OIR. Fasting glucose and insulin concentrations from the infusion study were used for the calculation of HOMA-IR. Clinical characteristics of the study group are shown in the Table.

**Table.** Clinical Characteristics and Study Measurements in Cohort Grouped by Presence of Obesity and Insulin Sensitivity

Clinical features	Lean		OIS		OIR	
	Participants, No.	Measurement, median (IQR)	Participants, No.	Measurement, median (IQR)	Participants, No.	Measurement, median (IQR)
Age, y	18	16.5 (15.8-18.0)	12	18.0 (16.0-20.0)	14	18.5 (15.8-20.0)
Sex, No. (%)	18		12			
Female		8 (44)		8 (66)	14	9 (64)
Male		10 (56)		4 (33)	14	5 (36)
Race and ethnicity, No. (%)	18		12			
African American		0		2 (17)	14	8 (57)
Asian		1 (5)		0	14	0
Hispanic		1 (5)		5 (42)	14	4 (29)
White		16 (89)		5 (42)	14	2 (14)
Body mass index^a^	18	22.0 (20.1-23.6)	12	31.8 (27.9-35.0)	14	41.2 (36.2-47.0)
Body fat, %	18	16.8 (12.2-22.5)	12	39.0 (29.0-41.9)	13	48.6 (42.2-53.5)
Fasting plasma insulin, μU/mL	18	11.1 (8.96-14.8)	11	12.4 (7.56-14.7)	13	36.7 (29.7-41.8)
Fasting plasma glucose, mg/dL	18	87.0 (85.0-91.3)	12	86.0 (80.3-93.0)	13	95.0 (90.0-101)
Fasting plasma C-peptide, ng/mL	18	2.06 (1.43-2.45)	11	2.01 (1.64-2.58)	13	3.35 (2.71-4.37)
HOMA-IR	18	2.39 (1.85-3.22)	12	2.77 (1.92-3.72)	14	8.78 (6.64-10.0)
WBISI	10	5.01 (4.63-10.2)	11	3.61 (3.44-4.89)	12	1.42 (0.97-1.68)
Disposition index	10	10.3 (5.98-13.2)	11	9.86 (3.84-14.5)	12	4.42 (2.94-6.57)
Basal H_2_, ppm	17	22.0 (13.5-31.5)	11	16.0 (2.00-22.0)	14	28.0 (4.00-36.2)
CH_4_, No. (%)	18		12			
Producer		5 (28)		1 (8)	14	4 (29)
Nonproducer		13 (72)		11 (92)	14	10 (71)
Plasma acetate enrichment at basal steady state (120-180 min), MPE	18	10.4 (6.13-17.9)	10	13.4 (6.65-20.8)	11	14.1 (9.50-15.4)
Plasma acetate enrichment at postlactulose steady state (420-600 min), MPE	18	5.40 (4.30-8.90)	10	7.65 (5.43-9.98)	11	9.70 (8.90-9.90)
Basal Ra_acetate_, μmol × kg^−1^ × min^−1^	18	6.39 (3.53-10.8)	10	4.68 (2.68-12.1)	11	4.74 (4.27-8.32)
Fasting plasma FFA, mM	18	0.535 (0.447-0.726)	11	0.454 (0.253-0.591)	14	0.512 (0.43-0.64)
Basal PYY, pg/mL	18	135 (117-146)	12	141 (98.0-166)	14	117 (108-136)
Basal ghrelin, pg/mL	18	902 (631-1190)	12	782 (554-849)	14	494 (381-551)
Basal GLP-1, pg/mL	18	15.9 (8.01-41.8)	12	7.33 (2.32-22.7)	12	6.12 (3.04-35.8)
Basal ATIS, index	18	0.30 (0.23-0.37)	11	0.27 (0.21-0.42)	13	0.09 (0.08-0.14)

Abbreviations: BMI, body mass index; CH_4_, methane; FFA, free fatty acid; GLP-1, Glucagon-like peptide-1; HOMA-IR, Homeostatic Model Assessment of Insulin Resistance; H_2_, hydrogen; MPE, mole percent excess; OIR, Obese Insulin Resistant; OIS, Obese Insulin Sensitive; PYY, peptide YY; WBISI, Whole Body Insulin Sensitivity Index.

SI conversion factors: To convert insulin to pmol/L, multiply by 6.945; to convert glucose to mmol/L, multiply by 0.0555; to convert C-peptide to nmol/L, multiply by 0.331; to convert tyrosine to μmol/L, multiply by 55.19; to convert glucagon to ng/L, multiply by 1.0.

^a^ Body mass index is calculated as weight in kilograms divided by height in meters squared.

### Outcomes and Hypotheses

The primary outcome of the study was the response of PYY, ghrelin, active GLP-1, and FFA to the colonic fermentation of 20 g of lactulose. Secondary outcomes include responses in insulin secretion and clearance; adipose tissue insulin sensitivity; and colon derived hydrogen, methane, and acetate production. These parameters were evaluated in the whole group as well as in the subgroups that were created by categorizing the participants according to the presence of obesity and insulin resistance. We hypothesized that lean, OIS, and OIR would experience a different response to colonic fermentation; that the previously observed difference in acetate synthesis between lean youth and youth with obesity may be exacerbated by insulin resistance (primary outcome); and that the 3 groups studied may show different changes in PYY, ghrelin, and active GLP-1 concentrations as well as FFA and adipose tissue insulin resistance (secondary outcomes) in response to the colonic fermentation of 20 g of lactulose. A detailed description of the experimental design, study procedures, and statistical analysis can be found in eMethods in Supplement 1.

### Statistical Analysis

Data are reported as median (IQR) for continuous variables and count (percentage) for categorical variables. Differences between the 3 groups were compared using a Kruskall-Wallis test. Differences between 2 groups were compared using a Mann Whitney U test. χ^2^ tests were used to compare group differences for categorical data. Deltas (Δ) for PYY, ghrelin, active GLP-1, FFA, ISR, and insulin clearance were calculated by subtracting the peak or nadir value of the hormone for each participant from the concentration at 180 minutes. For FFA and adipose tissue insulin resistance, since there was an early change occurring already at 180 minutes, the basal concentration was at 120 minutes. Statistical significance was established at an α of 0.05 and *P*-values between 0.05 and 0.10 were considered a trend. Statistical analyses and graphs were generated using GraphPad Prism9 software version 9.0.0 for macOS (GraphPad Software).

## Results

### Clinical Characteristics of Study Participants

Forty-four participants completed the study and were included in the final data analysis (median [IQR] age, 17.5 [16.0-19.3] years; 25 [56.8%] were female; 23 [52.3%] were White). The clinical characteristics of the study cohort are shown in the Table. Participants were similar in terms of age and sex distribution, however, the groups differed by race and ethnicity, grouped by White, Hispanic, African American, Asian, and by BMI, and percent body fat (Table). Lean and OIS had a comparable degree of insulin sensitivity (HOMA-IR median [IQR]: lean, 2.39 [1.85-3.22]; OIR, 2.77 [1.92-3.72]; *P* = .49) despite having different mean BMI and percent body fat, while OIR had a more severe degree of IR (OIR median [IQR], 8.78 [6.64-10.0] compared with lean and OIS (*P* < .001 and *P* < .001, respectively). Median (IQR) adipose insulin sensitivity (ATIS) at baseline was significantly lower in the OIR group (0.097 [0.08-0.14]) as compared with the OIS (0.27 [0.208-0.419]; *P* = .004) and lean (0.30 [0.23-0.37]; *P* = .002) groups, but there was no difference between OIS and lean (*P* = .98).

### Association Between Insulin Resistance in Youth With Obesity and Rate of Acetate Appearance

**Figure 1.**
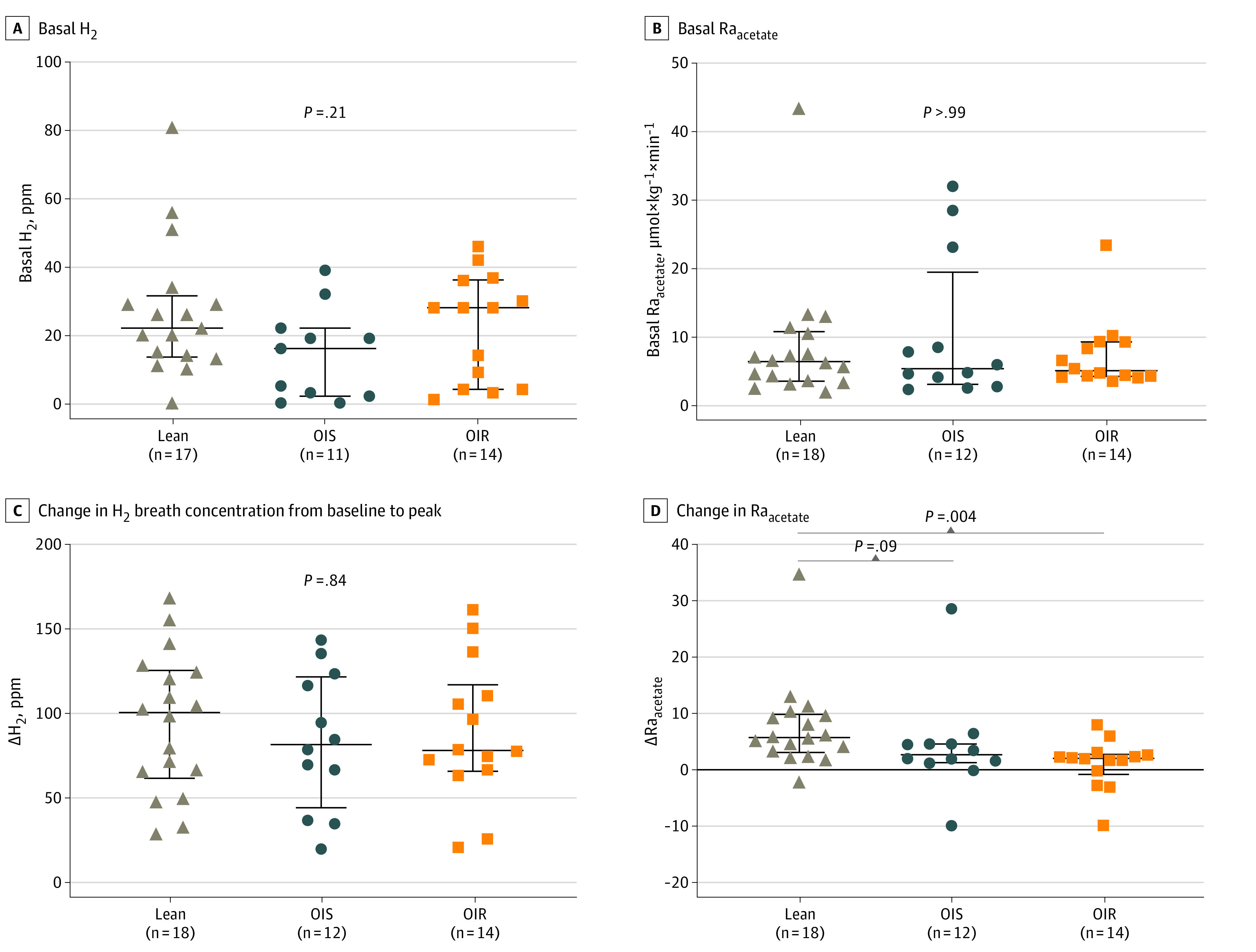
Measurement of Products of Colonic Fermentation in Lean, OIS, and OIR Participants The graphs show changes from baseline steady state to post-lactulose steady state in lean, OIS, and OIR subjects. *P* values comparing the three groups are from a Kruskall-Wallis test. Comparisons between 2 groups are from a Mann-Whitney *U* test. Error bars represent IQR. Δ indicates change; H_2_, hydrogen; Ra_acetate_, rate of acetate appearance.

Hydrogen production and rate of acetate appearance (Ra_acetate_) increase after lactulose ingestion consequent to colonic fermentation.^12^ When the groups were compared, there was a similar basal hydrogen production and basal Ra_acetate_ between lean, OIS, and OIR (Figure 1A-B). After the ingestion of lactulose, the increase in hydrogen production was similar between the 3 groups (*P* = .84), while the median (IQR) increase in Ra_acetate_ following intestinal fermentation was higher in lean (5.69 [3.04 to 9.77] μmol × kg^−1^ × min^−1^) as compared with OIS (2.63 [1.22 to 4.52] μmol × kg^−1^ × min^−1^; *P* = .09) and OIR (2.00 [−0.86 to 2.69] μmol × kg^−1^ × min^−1^; *P* = .004) (Figure 1C-D). Methane production was measured in the subgroups and not all participants were methane producers; however, there was a similar rate of methane producers vs nonproducers between the groups (Table).

### Improvements in ATIS and Insulin Resistance as a Consequence of Colonic Fermentation

**Figure 2.**
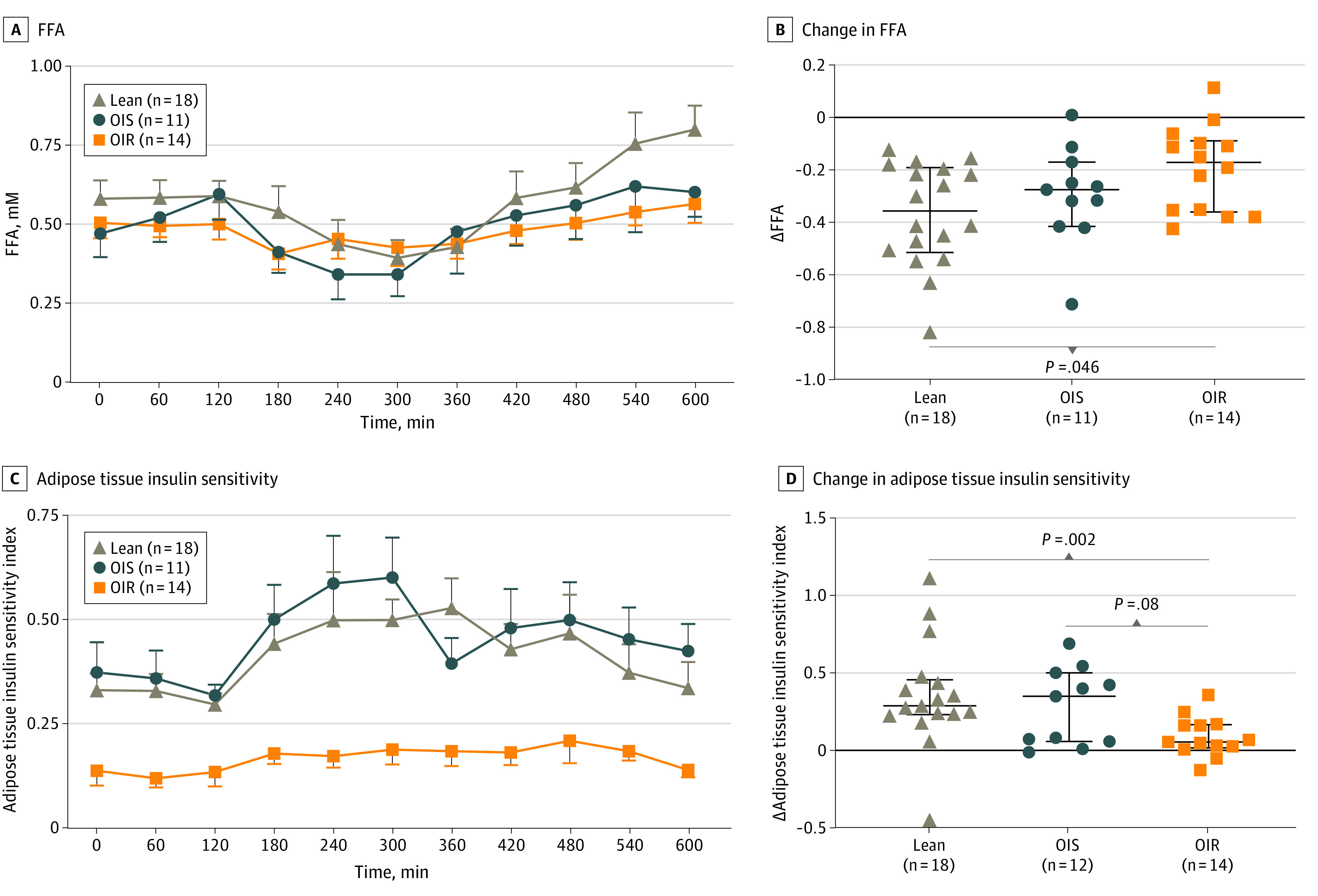
Measurement of Lipolysis and Adipose Tissue Insulin Sensitivity in Lean, OIS, and OIR Participants Comparisons between 2 groups are from a Mann-Whitney *U* test. Adipose tissue insulin sensitivity index (ATIS) is a surrogate index of insulin sensitivity to circulating FFA and was obtained by using the following formula: 2/((fasting insulin [mU/L] × fasting FFA [mmol/L]) + 1). Error bars represent IQR. Δ indicates change; FFA, free fatty acids.

**Figure 3.**
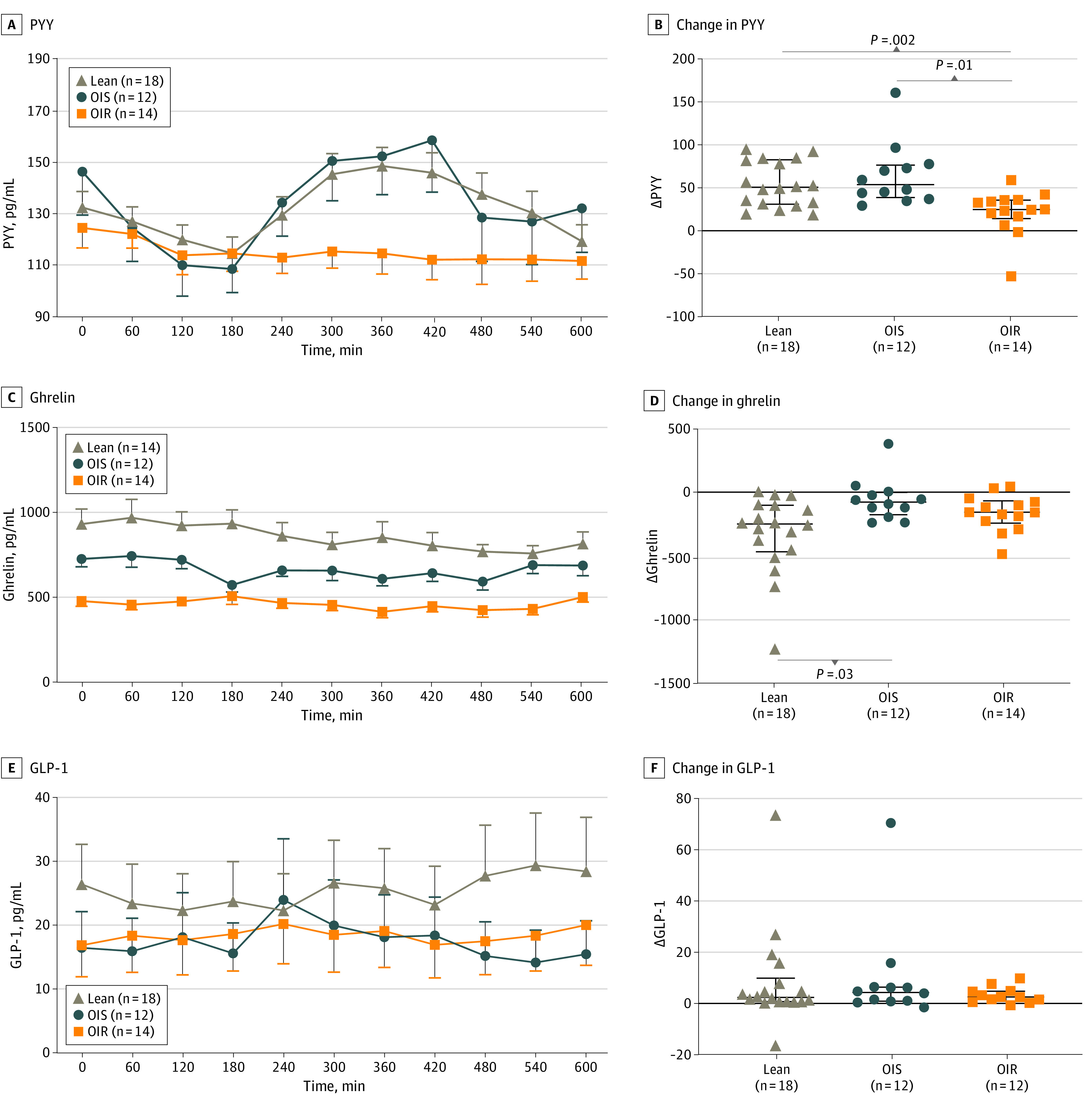
Measurement of Gut-Derived Hormones in Lean, OIS, and OIR Participants Comparisons between 2 groups are from a Mann-Whitney *U* test. Error bars represent IQR. Δ indicates change; GLP-1, active glucagon-like peptide 1; PYY, peptide tyrosine tyrosine.

FFA plasma concentration declines after colonic fermentation occurs (Figure 2A). Fasting concentrations of FFA were not different between the groups (Table). Median (IQR) changes in FFA from 120 minutes to the nadir were more marked in lean (−0.35 [−0.51 to −0.19] mM) as compared with OIR (−0.27 [−0.41 to −0.17] mM; *P* = .05) (Figure 3B). Basal ATIS was significantly different among the groups (Table) and increased in lean and OIS participants after lactulose ingestion, with peak sensitivities at 300 and 360 minutes in lean and OIS (Figure 3C), respectively. The groups showed different responses in ATIS after lactulose ingestion, in which lean and OIS showed an improvement, whereas OIR showed a minimal response (Figure 3C-D). Lean and OIS showed a greater median (IQR) increase in ATIS (lean: 0.28 [0.22 to 0.45]; OIS: 0.34 [0.05 to 0.49]) as compared with OIR (0.04 [0.01 to 0.16]; *P* = .002 and *P* = .08, respectively), but there was no difference between lean and OIS (Figure 3D). Changes in hydrogen during the study (baseline to peak production after lactulose ingestion) were not associated with decline in FFA in the whole cohort (*r*^2^ = 6.21 × 10^-7^; *P* = .99).

### Changes in PYY, Active GLP-1, and Ghrelin Consequent to Colonic Fermentation and Association With Insulin Resistance

PYY, active GLP-1, and ghrelin concentrations at 0 minutes are shown in the Table. The changes in plasma concentrations of enteroendocrine peptides after the ingestion of lactulose are shown in Figure 3. PYY concentrations increased after lactulose ingestion, with peak concentrations at 360 minutes in lean and at 300 minutes in OIS (Figure 3A). Median (IQR) ΔPYY between 180 minutes and peak concentration was similar between lean (51.3 [31.6 to 83.3] pg/mL) and OIS (54.3 [39.3 to 77.2] pg/mL) (*P* = .99), but the ΔPYY in the OIR group (25.4 [14.8 to 36.4] pg/mL) was significantly lower than lean (*P* = .002) and OIS (*P* = .01) (Figure 3B). Plasma concentrations of ghrelin had a modest decline in the subgroups after lactulose ingestion, with lean showing the greatest median (IQR) decrease (−238 [−458 to −88.3] pg/mL) and OIS and OIR showing less marked reductions (OIS, −63.5 [−163 to 12.6] pg/mL; OIR, −145 [−231 to −55.0] pg/mL) over the study (Figure 3C). Comparison of Δghrelin concentration showed significant differences between lean vs OIS group (*P* = .01), but no differences were found for OIR compared with other groups (OIR vs lean: *P* = .337; OIR vs OIS: *P* = .61) (Figure 3D). Active GLP-1 concentrations during the study and ΔGLP-1 between 180 minutes and peak concentration were similar between the lean, OIS, and OIR groups (*P* = .83) (Figure 3E-F). Changes in hydrogen concentration during the study (baseline to peak production after lactulose ingestion) were not associated with changes in PYY (*r*^2^ = 0.007; *P* = .58), ghrelin (*r*^2^ = 0.003; *P* = .72), or active GLP-1 (*r*^2^ = 0.002; *P* = .41).

### Insulin Secretion and Clearance After Colonic Fermentation

Insulin secretion rate and clearance during the study in lean, OIS, and OIR are shown in eFigure 1 in Supplement 1. In the OIR group, the median (IQR) basal insulin secretion rate (ISRb) was higher (149 [119-193] pmol × min^−1^ × m^−2^) and the median (IQR) basal insulin clearance rate (Clinsb) was lower (0.67 [0.60-0.89] L × min^−1^ × m^−2^) as compared with the lean (ISRb: 103 [71.5-121] pmol × min^−1^ × m^−2^, *P* = .007; Clinsb, 1.10 [1.03-1.79] L × min^−1^ × m^−2^; *P* < .001) and OIS groups (ISRb, 99.9 [84.5-115] pmol × min^−1^ × m^−2^; *P* = .03; Clinsb, 1.31 [0.91-2.11] L × min^−1^ × m^−2^; *P* = .005) (eFigure 2 in Supplement 1). During the study, for the OIR group, total median (IQR) area under the receiving operator characteristic curve (AUC) for insulin secretion rate was higher (71.0 [61.4-88.3] nmol × m^−2^) and total AUC for insulin clearance rate was lower (0.719 [0.67-0.919] L × min^−1^ × m^−2^) as compared with lean (AUC_ISR_, 41.5 [30.6-53.6] nmol × m^−2^; *P* = .003; AUC_Clins_, 1.22 [0.93-1.50] L × min^−1^ × m^−2^; *P* = .001) and OIS (AUC_ISR_, 45.2 [32.5-51.2] nmol × m^−2^; *P* = .05; AUC_Clins_, 1.28 [1.16-1.52] L × min^−1^ × m^−2^; *P* = .001) groups (eFigure 2 in Supplement 1). ΔISR and Δinsulin clearance were numerically greater, though not statistically different, in the OIR group as compared with that in lean and OIS (eFigure 2 in Supplement 1). There was no association between Ra_acetate_ and basal insulin clearance in the whole group (*r*^2^ = 0.003; *P* = .77).

## Discussion

In this cross-sectional study, we found that (1) after the oral ingestion of lactulose, colonic fermentation occured to the same extent in youth with lean, OIS, and OIR phenotypes, but the increase of the rate of acetate appearance was lower in OIR as compared with the other groups; (2) adipose tissue insulin sensitivity improved after lactulose ingestion, but this improvement was blunted in OIR; and (3) lactulose ingestion elicited an enteroendocrine response that was characterized by an increase in PYY and active GLP-1 and a decline in ghrelin, but PYY and ghrelin response may be affected by degree of adiposity and insulin resistance. Consistent with previous findings in adults,^15^ our data show that colonic fermentation may not be associated with changes in insulin secretion in youth. In fact, a study by Petersen et al^15^ has shown that insulin secretion rates measured by using a hyperglycemic clamp do not induce higher insulin secretion.^15^ Furthermore, for the first time, we explored the association between colonic acetate production and insulin clearance and found no association between these 2 parameters.

We previously showed a difference in changes of Ra_acetate_ between lean youth and youth with obesity after lactulose ingestion.^12^ Herein, we further expand on this, finding that within the group of youth with obesity, those with a higher degree of insulin resistance show a smaller increase in the rate of appearance of acetate after lactulose, despite similar hydrogen production among the groups. Whether there is a lower production or increased first-pass hepatic uptake of acetate is difficult to determine. Our data suggest that since the degree of fermentation is similar among the groups, the production of acetate may also be similar, however, more colon-derived acetate may be taken up by first pass in the livers of insulin resistant individuals with a lower appearance in plasma. If this is the case, it would be important to understand the metabolic pathways toward which colonic acetate is being diverted in these individuals.

Changes in FFA and adipose tissue insulin sensitivity suggest a connection between colonic fermentation and adipose tissue lipolysis. Previous studies in healthy volunteers using D5-glycerol to measure changes in glycerol turnover, a strong proxy of adipose tissue lipolysis, have shown that there is suppression of adipose tissue lipolysis after lactulose ingestion.^16^ Similarly, in our study, we observe a reduction of FFA and an improvement of adipose tissue insulin sensitivity. The latter index suggests that when colonic fermentation occurs, adipose tissue responds better to the antilipolytic effects of insulin. It is of note that changes in FFA and adipose tissue insulin sensitivity occurred already at 180 minutes when lactulose was given to the patients. This would suggest that something else may initiate the process. The early fall in FFA concentration that is observed beginning at 120 minutes may be due to prolonged fasting.

In this study, we also observed an effect of colonic fermentation on the enteroendocrine response, involving PYY, ghrelin, and active GLP-1. In vitro and animal studies have shown that the effect that colonic fermentation has on adipose tissue may be mediated by the production of SCFAs in the gut. These metabolites bind free fatty acid receptors FFAR2 and FFAR3 on the L-cells of the gastrointestinal tract and cause the release of GLP-1 and PYY.^17,18^ The responses of PYY, active GLP-1, and ghrelin to lactulose ingestion suggest a positive effect of colonic fermentation on the enteroendocrine regulators of appetite.

A recent study by Christiansen et al^19^ showed that the colonic fermentation of lactulose did not influence the concentrations of GLP-1 or PYY in healthy young men, as their group found that concentrations of PYY and GLP-1 peaked much earlier than the production of hydrogen, which suggests that ingestion of lactulose caused hormone release in the small intestine likely due to increased osmolarity, previously shown by another study in humans.^19^ These results are different from our findings, as we observed that lean and insulin sensitive youth with obesity have a nadir FFA concentration at 300 minutes and peak PYY concentrations at 360 to 420 minutes, which reflects the concomitant rise of hydrogen during the study. In a randomized crossover trial^20^ in which 25 individuals were challenged with 75 g glucose, 24 g inulin, or 28 g resistant starch, neither inulin nor resistant starch were associated with changes in PYY and GLP-1.

Whether the effect is due to the byproducts of colonic fermentation or by the passage of the lactulose in the intestine, undigestible carbohydrates play an important role in modulating gastrointestinal signals of hunger and satiety. Interestingly, the responses of PYY and ghrelin are blunted when obesity and insulin resistance occur. These data suggest that once insulin resistance occurs, the release of intestinal satiety hormones in response to SCFAs may be impaired and that the benefit of a diet rich in indigestible fiber may be lost, even as early as in childhood (enteroendocrine inflexibility). These data are consistent with a previous observation that a 6-week treatment with inulin—an undigestible type of carbohydrate—does not improve PYY and GLP-1 secretion in adults with type 2 diabetes.^21^

### Limitations

We acknowledge that our study has some limitations, such as the small sample size of the groups, lack of dietary recall to assess previous fiber consumption in our cohort, and the lack of stable isotope studies to measure glycerol turnover, which is a better estimate of adipose tissue lipolysis vs the measurement of plasma FFA alone. Moreover, although we have been able to measure insulin sensitivity, secretion, and clearance, we were not able to investigate the association between colonic fermentation and beta cell glucose sensitivity. Nevertheless, our study has some important strengths, such as the thorough clinical phenotyping of our cohort, the use of stable isotope infusion to determine acetate turnover, the long-lasting experiment, measure of enteroendocrine hormones, and assessment of adipose tissue insulin resistance and insulin clearance.

## Conclusions

Our data suggest that the benefits derived from the metabolism of dietary fibers on adipose tissue and the enteroendocrine system may be lost in youth with obesity and insulin resistance. These findings shed light on the role that insulin resistance may have on this association.

## Notes

Supplement 1.
eMethods.
**eFigure 1. **Insulin Secretion and Clearance Over Time **eFigure 2. **Insulin Secretion and Clearance Among Lean, Obese Insulin Sensitive, and Obese Insulin Resistant Patients
eReferences

Supplement 2.
Data Sharing Statement
