# Supplementary material for: Comparison of Metabolic Response to Colonic Fermentation in Lean Youth vs Youth With Obesity
Source: JAMA Netw Open. 2023 May 9;6(5):e2312530. doi: 10.1001/jamanetworkopen.2023.12530 (PMC10170343; doi:10.1001/jamanetworkopen.2023.12530)
Supplement: Supplement 1. — eMethods. eFigure 1. Insulin Secretion and Clearance Over Time eFigure 2. Insulin Secretion and Clearance Among Lean, Obese Insulin Sensitive, and Obese Insulin Resistant Patients eReferences [file jamanetwopen-e2312530-s001.pdf]

Supplemental Online Content

Galuppo B, Umano GR, Li Z, et al. Comparison of metabolic response to colonic fermentation in lean youth vs youth with obesity. *JAMA Netw Open*. 2023;6(5):e2312530. doi:10.1001/jamanetworkopen.2023.12530

eMethods.
eFigure 1. Insulin Secretion and Clearance Over Time
eFigure 2. Insulin Secretion and Clearance Among Lean, Obese Insulin Sensitive, and Obese Insulin Resistant Patients
eReferences

This supplemental material has been provided by the authors to give readers additional information about their work.

© 2023 Galuppo B et al. *JAMA Network Open.*

eMethods

Experimental Design and Study Procedures

Participants completed a 3-hour 10-timepoint oral glucose tolerance test (OGTT) as previously described [1, 2] and a 10-hour sodium d3-acetate intravenous infusion study at the Hospital Research Unit (HRU) of Yale New Haven Hospital in New Haven, CT. Anthropometric measurements such as height, weight, and BMI were obtained at the OGTT visit. BMI percentile was calculated for each participant according to the Center for Disease Control (CDC) pediatric growth charts which account for age and sex. Race and ethnicity were self-reported by participants.

Study to Assess Acetate Turnover ($Ra_{acetate}$)

The sodium d3-acetate infusion studies have been previously described [3]. In brief, sodium d3-acetate was infused into peripheral circulation for three hours to reach steady state concentration, then at 180 minutes, participants ingested 20g of lactulose, an indigestible carbohydrate, to stimulate colonic fermentation. $Ra_{acetate}$ was calculated as previously shown[3] using the timepoints at which d3-acetate steady state was reached (e.g., averages of 120, 150, and 180 minutes for the pre-lactulose phase and 400 through 600 minutes for the post-lactulose phase)[3]. Delta (Δ) $Ra_{acetate}$ is calculated by subtracting the average $Ra_{acetate}$ during the post-lactulose phase from the average $Ra_{acetate}$ during the pre-lactulose phase[3].

Breath samples were collected during this study in glass tubes at baseline (0 minutes) and then hourly for the measurement of hydrogen (H_2), an indicator of colonic fermentation, and methane (CH_4). Breath collection and analysis have been previously described [3]. CH_4 data is reported categorically as “producer” or “non-producer,” with “producer” being defined as a production of CH_4 concentration greater than 6 ppm. Blood samples were obtained at -15 and 0

minutes and then hourly for the duration of the study for plasma concentration measurement of glucose, insulin, active glucagon-like peptide-1 (GLP-1), peptide YY (PYY), ghrelin, and free fatty acid (FFA). Adipose tissue insulin sensitivity (ATIS) was calculated from an OGTT as previously described [4]. The adipose insulin resistance index was calculated by obtaining the product of fasting insulin and FFA concentration.

Insulin secretion rate and Insulin clearance

Insulin secretion rate (ISR) was estimated by C-peptide deconvolution according to Van Cauter [5]. Insulin clearance was calculated as the ratio between ISR and plasma insulin [6-8]. Disposition index was calculated from the OGTT as the product of insulinogenic index and WBISI.

Biochemical Analysis

PYY, total ghrelin, and active GLP-1 were measured by an ELISA assay. Plasma glucose was measured at bedside during the OGTT and infusion studies using the YSI2700-STAT-Analyzer (Yellow Springs Instruments, Yellow Springs, OH). Plasma insulin and C-peptide were measured by antibody radioimmunoassay from Millipore Sigma (Billerica, MA). FFA were measured using standard automated kinetic enzymatic assays (Wake Chemicals Inc.).

Measurement of Colonic Production of H_2

Breath samples were collected at baseline (0 minutes) and hourly using 10 mL glass Exetainer vials (Labco Ltd, Lampeter, UK). Participants were instructed to exhale into the vial using a straw and then the vial was immediately closed with a rubber stopper. Samples were analyzed for H_2 concentrations using gas chromatography as previously described [3].

Bioelectrical Impedance Analysis

© 2023 Galuppo B et al. *JAMA Network Open.*

A TANITA digital scale was used to measure body fat percentage by bioelectrical impedance analysis in all participants at the OGTT visit.

Statistics

Data are reported as median (interquartile range) for continuous variables and count (%) for categorical variables. Differences between the three groups were compared using a Kruskall-Wallis test. Differences between two groups were compared using a Mann Whitney *U* test. Chi-squared tests were used to compare group differences for categorical data. Deltas (Δ) for PYY, ghrelin, active GLP-1, FFA, ISR, and insulin clearance were calculated by subtracting the peak or nadir value of the hormone for each subject from the concentration at 180 minutes. For FFA and adipose tissue insulin resistance, since there was an early change occurring already at 180 minutes, the basal concentration was at 120 minutes. Statistical significance was established at an alpha of 0.05 and P-values between 0.05 and 0.10 were considered a trend. Original data collected are available at the following link: https://doi.org/10.6084/m9.figshare.22138016.v2.

Statistical analyses and graphs were generated using GraphPad Prism9 software 9.0.0 for macOS (San Diego, CA, USA).

© 2023 Galuppo B et al. *JAMA Network Open.*

eFigure 1. Insulin Secretion and Clearance Over Time

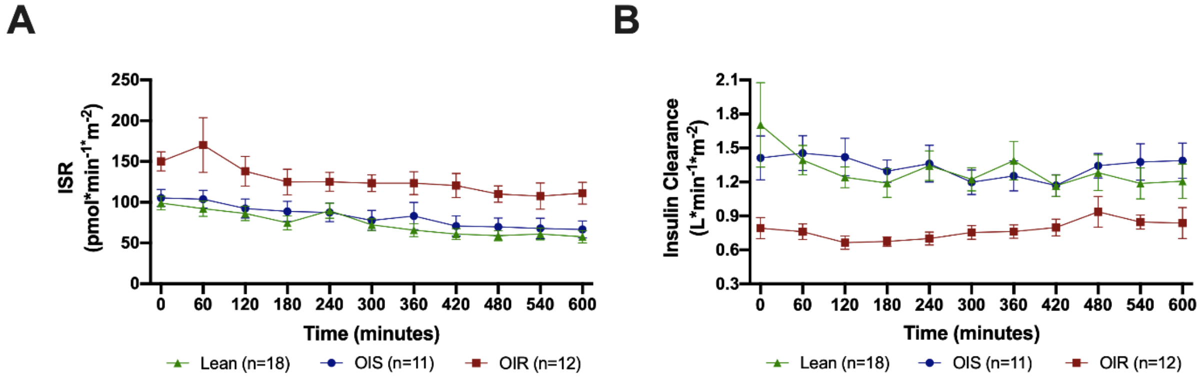

Supplemental Figure 1. A. Insulin secretion rate in Lean, OIS, and OIR during the study. **B.** Insulin clearance in Lean, OIS, and OIR during the study. Arrow indicates time of lactulose ingestion. ISR: insulin secretion rate.

© 2023 Galuppo B et al. *JAMA Network Open.*

eFigure 2. Insulin Secretion and Clearance Among Lean, Obese Insulin Sensitive, and Obese Insulin Resistant Patients

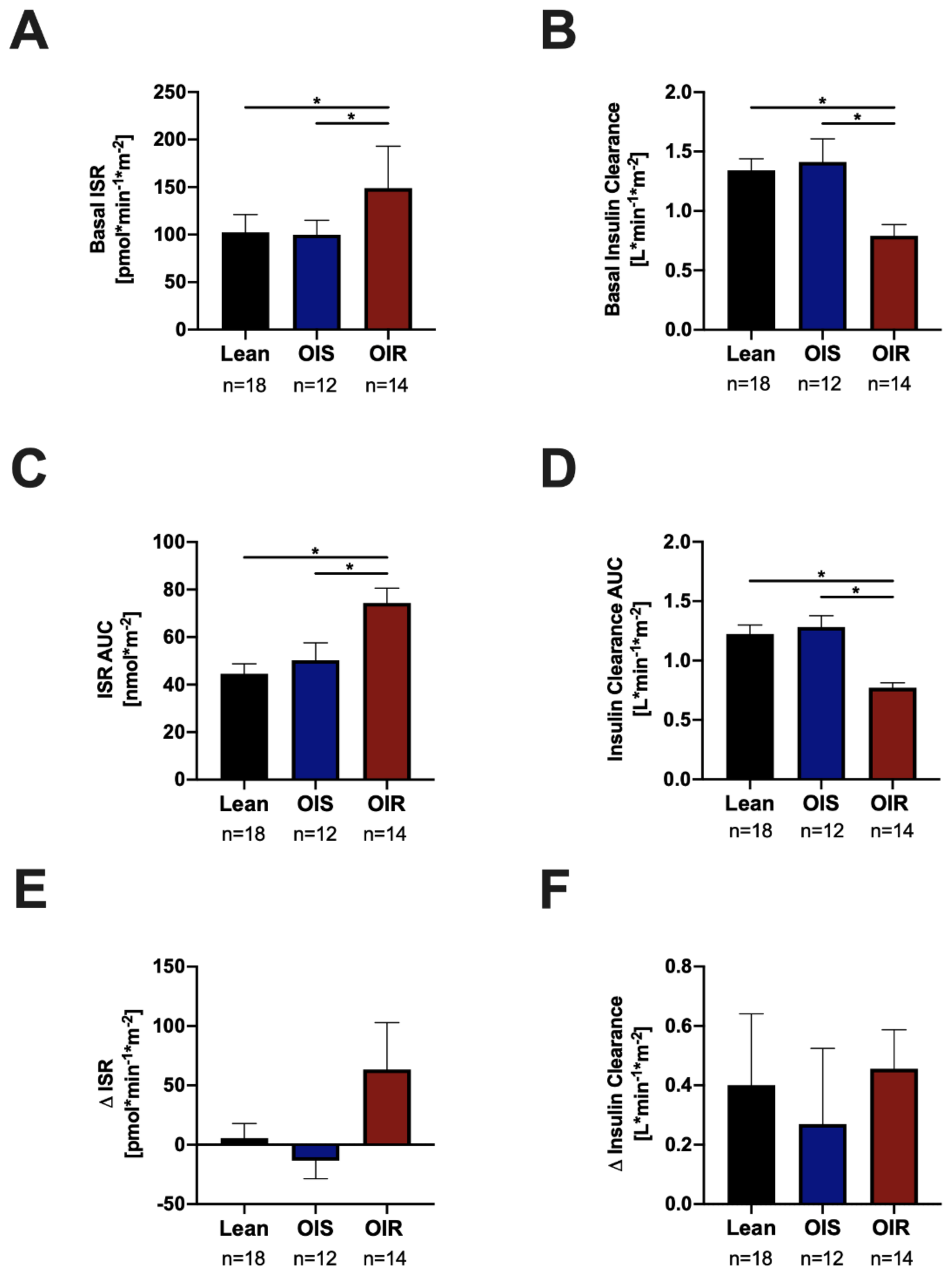

Supplemental Figure 2. A. Basal ISR is greater in OIR than in Lean and OIS. **B.** Basal insulin clearance is lower in OIR than in Lean and OIS. **C.** OIR showed a greater total ISR during the study than Lean and OIS. **D.** OIR showed a lower total insulin clearance during the study than Lean and OIS. **E**. Delta ISR and **F.** Delta insulin clearance in each group during the study. There was no difference in terms of delta ISR and delta insulin clearance among the groups. ISR: insulin secretion rate. P-values obtained by Kruskal-Wallis test. * indicates P<0.05.

© 2023 Galuppo B et al. *JAMA Network Open.*

eReferences

1. Holder T, Giannini C, Santoro N, et al. A low disposition index in adolescent offspring of mothers with gestational diabetes: a risk marker for the development of impaired glucose tolerance in youth. *Diabetologia*. Nov 2014;57(11):2413-20. doi:10.1007/s00125-014-3345-2
2. Weiss R, Magge SN, Santoro N, et al. Glucose effectiveness in obese children: relation to degree of obesity and dysglycemia. *Diabetes Care*. Apr 2015;38(4):689-95. doi:10.2337/dc14-2183
3. Galuppo B, Cline G, Van Name M, et al. Colonic Fermentation and Acetate Production in Youth with and without Obesity. *J Nutr*. Nov 2 2021;151(11):3292-3298. doi:10.1093/jn/nxab277
4. Hershkop K, Besor O, Santoro N, Pierpont B, Caprio S, Weiss R. Adipose Insulin Resistance in Obese Adolescents Across the Spectrum of Glucose Tolerance. *J Clin Endocrinol Metab*. Jun 2016;101(6):2423-31. doi:10.1210/jc.2016-1376
5. Van Cauter E, Mestrez F, Sturis J, Polonsky KS. Estimation of insulin secretion rates from C-peptide levels. Comparison of individual and standard kinetic parameters for C-peptide clearance. *Diabetes*. Mar 1992;41(3):368-77. doi:10.2337/diab.41.3.368
6. Tricò D, Galderisi A, Mari A, Santoro N, Caprio S. One-hour post-load plasma glucose predicts progression to prediabetes in a multi-ethnic cohort of obese youths. *Diabetes Obes Metab*. May 2019;21(5):1191-1198. doi:10.1111/dom.13640
7. Tricò D, Galderisi A, Mari A, et al. Intrahepatic fat, irrespective of ethnicity, is associated with reduced endogenous insulin clearance and hepatic insulin resistance in obese youths: A cross-sectional and longitudinal study from the Yale Pediatric NAFLD cohort. *Diabetes Obes Metab*. Sep 2020;22(9):1628-1638. doi:10.1111/dom.14076
8. Trico D, Galderisi A, Van Name MA, et al. A low n-6 to n-3 polyunsaturated fatty acid ratio diet improves hyperinsulinaemia by restoring insulin clearance in obese youth. *Diabetes Obes Metab*. Jul 2022;24(7):1267-1276. doi:10.1111/dom.14695

© 2023 Galuppo B et al. *JAMA Network Open.*
