# Supplementary material for: Comparison of Metabolic Response to Colonic Fermentation in Lean Youth vs Youth With Obesity
Source: JAMA Netw Open. 2023 May 9;6(5):e2312530. doi: 10.1001/jamanetworkopen.2023.12530 (PMC10170343; doi:10.1001/jamanetworkopen.2023.12530)
Supplement: Supplement 2. — Data Sharing Statement [file jamanetwopen-e2312530-s002.pdf]

Data Sharing Statement

Galuppo. Comparison of Metabolic Response to Colonic Fermentation in Lean Youth vs Youth With Obesity. *JAMA Netw Open*. Published May 09, 2023.
doi:10.1001/jamanetworkopen.2023.12530

Data
Data available: Yes
Data types: Deidentified participant data
How to access data: Original data collected are available at the following link:
https://doi.org/10.6084/m9.figshare.22138016.v2
When available: With publication

Supporting Documents
Document types: None

Additional Information
Who can access the data: anyone requesting the data
Types of analyses: for any purpose
Mechanisms of data availability: with investigator support
